# Air pollution and mobility patterns in two Ugandan cities during COVID-19 mobility restrictions suggest the validity of air quality data as a measure for human mobility

**DOI:** 10.1007/s11356-022-24605-1

**Published:** 2022-12-15

**Authors:** Ronald Galiwango, Engineer Bainomugisha, Florence Kivunike, David Patrick Kateete, Daudi Jjingo

**Affiliations:** 1 The African Center of Excellence in Bioinformatics and Data Intensive Sciences, The Infectious Diseases Institute, Makerere University, Kampala, Uganda; 2 Center for Computational Biology, Uganda Christian University, Mukono, Uganda; 3 Department of Computer Science, College of Computing and Information Sciences, Makerere University, Kampala, Uganda; 4 Department of Immunology and Molecular Biology, College of Health Sciences, Makerere University, Kampala, Uganda; 5 Department of Medical Microbiology, College of Health Sciences, Makerere University, Kampala, Uganda

**Keywords:** Air quality, Human mobility, Transmission, Infectious diseases, Particulate matter, COVID-19

## Abstract

We explored the viability of using air quality as an alternative to aggregated location data from mobile phones in the two most populated cities in Uganda. We accessed air quality and Google mobility data collected from 15^th^ February 2020 to 10^th^ June 2021 and augmented them with mobility restrictions implemented during the COVID-19 lockdown. We determined whether air quality data depicted similar patterns to mobility data before, during, and after the lockdown and determined associations between air quality and mobility by computing Pearson correlation coefficients ($R$), conducting multivariable regression with associated confidence intervals (CIs), and visualized the relationships using scatter plots. Residential mobility increased with the stringency of restrictions while both non-residential mobility and air pollution decreased with the stringency of restrictions. In Kampala, PM_2.5_ was positively correlated with non-residential mobility and negatively correlated with residential mobility. Only correlations between PM_2.5_ and movement in work and residential places were statistically significant in Wakiso. After controlling for stringency in restrictions, air quality in Kampala was independently correlated with movement in retail and recreation (− 0.55; 95% CI =  − 1.01– − 0.10), parks (0.29; 95% CI = 0.03–0.54), transit stations (0.29; 95% CI = 0.16–0.42), work (− 0.25; 95% CI =  − 0.43– − 0.08), and residential places (− 1.02; 95% CI =  − 1.4– − 0.64). For Wakiso, only the correlation between air quality and residential mobility was statistically significant (− 0.99; 95% CI =  − 1.34– − 0.65). These findings suggest that air quality is linked to mobility and thus could be used by public health programs in monitoring movement patterns and the spread of infectious diseases without compromising on individuals’ privacy.

**Supplementary information:**

The online version contains supplementary material available at 10.1007/s11356-022-24605-1.

## Introduction

Human mobility has previously been used as a proxy of infectious disease transmission especially of respiratory pathogens such as *Mycobacterium tuberculosis* (Aspler et al. 2010; Moreno et al. 2017; Robsky et al. 2021) and influenza (Engebretsen et al. 2020) and other diseases such as malaria (Milusheva 2020). More recently, the COVID–19 pandemic has emphasized the role of mobility in disease transmission (Badr et al. 2020; Chen et al. 2020; Kartal et al. 2021; K. Liu et al. 2020a, b; Rahman et al. 2021; Yang et al. 2020). A virus that reportedly originated from Wuhan, China (Y.-C. Liu et al. 2020a, b), found its way out of the city, spread throughout China, and was exported to other countries with which China shares a physical border and others overseas, starting with sporadic cases in many countries and thereafter a widespread community transmission. Thus, on 11^th^ March 2020, the World Health Organization (WHO) declared COVID-19 a global pandemic. Mobility data have aided infectious disease control programs to identify transmission hotspots and consequently informed targeted interventions. Mobility data have also been used to derive contact matrices that are used to parametrize mathematical models used in predicting COVID-19 infections that could be observed and to assess the effectiveness of interventions (Prem et al. 2020; 2021).

To date, aggregated location data from mobile phones have been the main means to capture mobility patterns of individuals at the population level (Badr et al. 2020). However, mobile phone location data come with various ethical challenges relating to privacy and are thus aggregated to ensure anonymity. Its other limitations include the relative paucity of mobile phones with enabled Global Positioning System (GPS) location devices in developing countries (Kibuacha 2021), the ability of users to turn off GPS location, and the rather arduous and lengthy process of obtaining such data from mobile phone companies. This renders them less useful in studying infectious disease transmission. An estimated 16% and an average of 30% of people have a smart phone in Uganda and sub-Saharan Africa, respectively (Kibuacha 2021). Furthermore, air quality data provides better resolution. For example, on top of having them ground stationed, low-cost air quality sensors developed by the AirQo project (Okure et al. 2022; Coker et al. 2021) have been mounted on boda-bodas (local motorbike transport) and commuter taxis whose ubiquity and agility facilitate more diversified and localized measurements. These two are the most used means of transport by the vast majority of people in Uganda.

Being less encumbered by the above listed limitations, ground monitored ambient air quality data could thus provide a viable alternative for understanding mobility patterns at population level. Air pollution in most cities is mainly due to vehicular movement and fumes from factories. Although the correspondence between human vehicular powered mobility and ambient particulate matter is in part intuitive, it has not been comprehensively tracked and studied using objective quantitative spatial–temporal metrics. This is a particularly valuable approach in the context of public health which has in recent times largely depended on mobile phone tracking to quantify human movement that could be related to disease spread. Air quality worsens as people move from their residences to their destinations such as places of work, recreation centers, and grocery shops and becomes better as movement lessens. Indeed, air quality improved during COVID-19 lockdowns around the world (Sannigrahi et al. 2021; Yechezkel et al. 2021; Wijnands et al. 2022; Naseer et al. 2022; Benchrif et al. 2021; Mishra et al. 2021; Broomandi et al. 2022; Arora et al. 2020; Li et al. 2022; Khan et al. 2021; Nicolini et al. 2022; Marinello et al. 2021; Bar et al. 2021), with most gains in air quality lost when the lockdowns were lifted, indicating that air quality could depict mobility patterns of individuals.

## Literature review

We conducted a literature review in which we searched PUBMED database for the most recent published literature on the association between quantitative measures of mobility and air quality, filtering for all published articles since the first reported case of COVID-19 globally. Literature shows that there is a growing number of studies showing the association between quantitative measures of mobility and air quality. For instance Cárcel-Carrasco et al. (2022) analyzed Apple mobility trend data (both driving and walking) as well as nitrogen dioxide (NO_2_) pollution levels in the cities of Tsuen Wan from Hong Kong (China), Los Angeles (USA), London (UK), São Paulo (Brazil), Bangalore (India), Johannesburg (South Africa), and Sydney (Australia) before, during, and after the COVID-19 lockdowns instituted in each city. There was an overall reduction in driving and walking in most of the cities during the lockdown compared with the pre-lockdown period. NO_2_ levels in Tsuen Wan in 2020 reduced compared to usual levels. Post lockdown, there was an increase in mobility but no increase in air pollution followed for all cities. Fan et al. (2020) investigated the impacts of traffic mobility reduction on PM_2.5_ (fine particulate matter) and PM_10_ (coarse particulate matter) concentrations in China with mobility patterns estimated from the intensity of residents’ traffic travel of a city. They found that PM_2.5_ and PM_10_ declined with reduction in traffic mobility, but this relationship varied spatially during the COVID-19 outbreak. Noda et al. (2021) investigated the relationship between reduction in population movement (quantified by the “social isolation index” created by the São Paulo State Government, as a yardstick for adopting official measures to fight COVID-19) in the city and the emission levels of PM_10_, PM_2.5_, No_x_ (nitrogen oxides), NO (nitric oxide), NO_2_, SO_2_ (sulfur dioxide), and CO (carbon monoxide). In their study, air pollution decreased with adherence rate to social isolation. Chang et al. (2021) investigated the impact of COVID-19 on air pollution in the two largest cities in Taiwan, which were not subject to economic or mobility restrictions. They found a shift in mode of transport from metro ridership to motor vehicle use during the COVID-19 period. This coincided with significant reductions in the levels of NO_2_ and PM_10_ during non-working days. Mohajeri et al. (2021) found a significant correlation between the lowering of NO_2_ levels and reduction in public transport ($p < 0.05$) and driving ($p < 0.05$) during the COVID-19 lockdown period in Greater London, Cardiff, Edinburgh, and Belfast. They also found air pollution at a lower level than expected as a result of the mobility restrictions imposed by the COVID-19 lockdowns. Efe (2022) compared mobility patterns (based on Google and Apple mobility data) and PM_10_, PM_2.5_, and NO_2_ concentrations during the COVID-19 lockdown period with the same periods during non-pandemic years, in Marmara Region, Turkey. The author found that PM_2.5_ and NO_2_ concentrations during the lockdown period declined with respect to the pre-lockdown period and the previous year. Overall, air quality worsened with increase in mobility. Sokhi et al. (2021) conducted a global analysis of changes in a variety of air quality species and mobility as a result of mobility restriction during the lockdown using four mobility databases: Apple driving, Google retail, Waze, and Baidu. They found reductions in air pollution in most cities as a result of the lockdown. Changes in mobility varied largely across cities and lockdown phases, with the greatest reductions observed during the full lockdown. Changes in NO_x_ concentrations were positively correlated with change in mobility. Ghahremanloo et al. (2022) investigated the impact of COVID-19 lockdowns on changes in PM_2.5_ concentrations in eleven metropolitan areas across the United States: Washington DC, New York, Boston, Chicago, Los Angeles, Houston, Dallas, Philadelphia, Detroit, Phoenix, and Seattle. They used Google Community Mobility Reports to study people’s mobility changes during the study period. They found reductions of PM_2.5_ in regions with larger decreases in mobility and those in which individuals remained in their residential areas longer. Pagsuyoin et al. (2022) computed correlations between CO, NO_2_, O_3_ (ozone), SO_2_, PM_2.5_, and PM_10_ concentrations in five metropolitan areas in the United States of America (USA) and mobility data obtained from Google’s Community Mobility Reports. They found that air quality was correlated with mobility but the direction of the relationship varied by city. Gorrochategui et al. (2021) investigated changes in NO_2_, O_3_, and PM_10_ in Barcelona metropolitan area and other parts of Catalonia during the COVID-19 lockdown with respect to pre-lockdown and to previous years (2018 and 2019). They computed correlations between air quality and mobility data obtained from Google’s Community Mobility Reports. In their study, correlation between mobility and air pollution was positive for NO_2_ and PM_10_ and negative for O_3_. Orak and Ozdemir (2021) investigated correlations between SO_2_ and mobility using data obtained from Google’s Community Mobility Reports. They found positive correlations between SO_2_ with restaurant/café mobility ($R = 0.32$, $p < 0.001$), transit mobility ($R = 0.26$, $p < 0.001$), and workplace mobility ($R = 0.39$, $p < 0.001$) and a negative correlation with stay-at-home ($R = -0.16$, $p = 0.007$). Zhu et al. (2020) computed correlations between human movements (mobile phone data from Baidu location-based services) and concentration of air pollutants. In their study, human mobility index was positively correlated with PM_2.5_ ($R = 0.06$, $p < 0.05$), PM_10_ ($R = 0.08$, $p < 0.05$), NO_2_ ($R = 0.05$, $p < 0.05$), and CO ($R = 0.04$, $p < 0.05$) and negatively correlated with O_3_ ($R = -0.04$, $p < 0.05$) and SO_2_ ($R = -0.03$, $p > 0.05$). It is evident from the above review that majority of the studies above have been conducted in cities of developed economies with the exception of a few (see, e.g., Noda et al. 2021; Cárcel-Carrasco et al. 2022).

In this study, we explored whether air quality data could be a viable alternative to aggregated location data from mobile phones, as a measure of human mobility using air quality and Google mobility data (Google LLC 2021) from the two most populated cities in Uganda, i.e., Kampala and Wakiso (UBOS 2019), for the period 15^th^ February 2020 to 10^th^ June 2021. It is this period that spanned the period before, during, and after the first major lockdown for both mobility and air quality data. As of 15^th^ October 2022, Community Mobility Reports are no longer being updated. We augmented these data with various COVID-19 restrictions implemented during this period including the timing of their institution and when they were relaxed or lifted to enable us to study air pollution and mobility patterns before, during, and after the lockdown. Prior to lockdown, economic activity which is largely characterized by vehicular movement was booming globally. However, as COVID-19 struck, economies shut down and movement reduced as restrictions on movement were implemented (Li et al. 2022). As lockdowns were lifted, economies started booming although some activities have never returned to pre-COVID-19 levels. We aimed to determine how mobility patterns were affected by COVID-19 mobility restrictions in two urban cities in Uganda and examine if air quality data depicted similar patterns. To quantify COVID-19 mobility restrictions, we made use of the government response stringency index—a composite measure of the strictness of policy responses (such as mobility restrictions) over time (Hale et al. 2021). We analyzed mobility data as well as the air quality data, determined how mobility restrictions impacted both mobility and air quality patterns, and determined the association between human mobility and air quality. The results from this study could provide infectious disease control programs with a high-resolution tool for studying human movement patterns and thus enable near-real time tracking of infectious disease spread without compromising on the privacy of individuals while avoiding several other limitations that come with the use of aggregated location data from mobile phones.

## Methodology

### Study setting

In order to study the association between human mobility and air quality, we needed three things, i.e., (1) human mobility data, (2) high-resolution air quality data that actually depicts movement of persons and is less associated with single point pollution events concentrated at factories, and (3) a control variable that affects both human mobility and air quality so as to determine if both mobility and air quality data depict similar patterns in reaction to changes in the control variable. The emergence of COVID-19 and its related restrictions on movement of persons presented a rare large-scale experimental control opportunity, to study and calibrate the relationship between human mobility and ambient air quality on an unprecedented scale. That opportunity coincided with the recent availability of community movement data from Google (Google LLC 2021).

Uganda was the ideal country to do this study because it implemented some of the most stringent mobility restrictions in the region and the world at large. For example, the country instituted a lockdown on 18^th^ March 2020 days before confirmation of her first case of COVID-19 on 21^st^ March 2021 and went on to suspend public gatherings, instituted a national-wide curfew, instituted stay-at-home and work-from orders, put restrictions on private and public transportation and on some days of the lockdown suspending both public and private transport except for health personnel and “essential services,” and maintained closure of educational institutions the longest in the world (The East African 2021; UNICEF 2021). This unique implementation of COVID-19 restrictions including instituting tight restrictions before a single case was confirmed enabled us to study air pollution and mobility patterns in the period before, during, and after the lockdown. Furthermore, this study draws insights from a developing economy context for which only a few similar studies have been conducted.

The cities of Kampala and Wakiso are found in the central region of Uganda and are part of the Kampala Metropolitan Area (KMA) which has become the country’s commercial, industrial, and education center with a highly mobile population commuting within, to, and from the area for trade, going for, and leaving workplaces and many students commuting to and from school. The area has experienced significant urban growth for many decades and was one of the fastest growing urban areas in Africa pre-COVID-19. Kampala is the capital, with a population of over 1.5 million people according to the Uganda Bureau of Statistics (UBOS 2019). Wakiso on the other hand partly encircles Kampala with the city headquarters lying approximately 12 miles, by road, northwest of Kampala, with a population of over 1.9 million people (UBOS 2019). Wakiso and Kampala are the first and second most populated districts in Uganda, respectively. Since the start of the COVID-19 pandemic, Google has availed human mobility data, in the form of Google Community Mobility Reports (Google LLC 2021), pertaining to movement in six kinds of places, i.e., grocery and pharmacy, parks, residential, retail and recreation, transit stations, and work places. Google mobility data for most districts in Uganda is sparse except for Kampala and Wakiso. This could be because these two most populated cities in the country are also urban centers with most smart phone users having phones with enabled GPS location devices being in these cities.

On top of having them ground stationed, low-cost air quality sensors developed by the AirQo project (Okure et al. 2022; Coker et al. 2021), an extensive air quality monitoring project that has a high-resolution network of low-cost air quality sensors, are mounted on boda-bodas (local motorbike transport) and commuter taxis whose ubiquity and agility facilitates more diversified and localized measurements. These two are the most used means of transport by the vast majority of people in Uganda. Thus, particulate matter detected by these sensors are an excellent representation of movement in this locality. Until 2022, which is outside the study period, when the project received funding from Google to expand across the entire country and in other African cities, most of these ground stationed air quality sensors and those mounted on boda-bodas and commuter taxis were located in Kampala and Wakiso.

### The data

#### Air quality data

AirQo (Okure et al. 2022; Coker et al. 2021) has deployed a number of air quality monitors across urban areas in Uganda, Kampala and Wakiso inclusive. Hourly data for air pollutant concentrations of particulate matter ≤ 2.5 μm and ≤ 10 μm (PM_2.5_ and PM_10_, respectively) for two cities (Kampala the capital and Wakiso her close neighbor) were obtained for the period 15^th^ February 2020 to 10^th^ June 2021. Across the entire available period, for each city, the 24-h average concentration (corresponding to a given day) for each pollutant was calculated. The average for each city was obtained by averaging overall air quality monitors (sensors) in each city.

#### Human mobility data

Human mobility data for Uganda was obtained from Google COVID-19 Community Mobility Reports (Google LLC 2021). Since the beginning of the COVID-19 pandemic, Google has been collecting data relating to change in visits to places classified as retail and recreation (places such as restaurants, cafés, shopping centers, theme parks, museums, libraries, and cinemas), supermarket and pharmacy (places such as supermarkets, food warehouses, farmers markets, specialty food shops, and pharmacies), parks (places like national parks, public beaches, marinas, dog parks, plazas, and public gardens), public transport (places that are public transport hubs, such as underground, bus, and train stations), workplaces (places of work), and residential (places of residence). These data are available as COVID-19 Community Mobility Reports that show how visits to different places change compared to a baseline. Changes for each day are compared to a baseline value for that day of the week. The baseline is the median value, for the corresponding day of the week, during the five-week period 3^rd^ January 2020–6^th^ February 2020. We obtained Google mobility data for the period 15^th^ February 2020 to 10^th^ June 2021.

We explored the possibility of using other sources of mobility data such as Apple mobility data. However, there is no Apple mobility data for Uganda just like for many countries in Africa (Cárcel-Carrasco et al. 2022). This is because most Ugandans (over 75%) are android phone users (UBOS, 2019). As of April 14^th^, 2022, Apple is no longer providing COVID-19 mobility trend reports.

#### COVID-19 lockdown measures instituted in Uganda during the study period

The government of Uganda instituted a lockdown on 18^th^ March 2021 days before confirmation of the first case of COVID-19 in the country on 21^st^ March 2021. This went on to be one of the toughest lockdowns in the region and the world at large. A report by the United Nations International Children’s Emergency Fund (UNICEF) stated that Uganda is one of the top countries that maintained closure of educational institutions the longest in the world (The East African 2021; UNICEF 2021). The lockdown included 32 days suspension of mass gatherings both political and cultural including public rallies, conferences, elections, cultural weddings exceeding 10 people, monthly markets, bars, music festivals, sports activities, cinemas, and concerts. All out-bound movement by Ugandans to or through category one countries (Italy, France, South Korea, China, USA, United Kingdom, Netherlands, Switzerland, Sweden, Belgium, Germany, Spain, Norway Austria, Malaysia, Pakistan, and San Marino) was also banned for 32 days. Non-agricultural work places including factories, hotels, large plantations, markets, and taxi-parks were allowed to continue functioning but with observance of Standard Operating Procedures (SOPs) stipulated by the country’s Ministry of Health. Burial was to be done by only nearby relatives. If the deceased was, however, suspected of dying from COVID-19, the state took over without the involvement of the family as the case is with Ebola victims.

All schools were closed for 30 days on 20^th^ March 2020 and the country’s international borders (air, land, or water) were closed on 21^st^ March 2020 except for cargo and goods following the confirmation of the first case. On the 25^th^ of March 2020, public transport (taxis, boats, buses, all passenger trains, and passenger motor cycles locally known as “boda-bodas” carrying passengers) was suspended for 14 days and private vehicles were restricted to carrying maximum 3 persons including the driver. Markets for non-food items including clothes, phones, and shoes were closed. Government offices were restricted to only “essential staff” while the non-essential staff were instructed to work from home except for those in critical agencies like the Uganda Revenue Authority, the country’s tax collection body. Following a surge in cases, all people movement was banned on 30^th^ March 2020 including those using private vehicles. Gatherings of more than 5 persons were also prohibited. All non-food shops like shopping malls, arcades, and hardware shops were closed for 14 days starting 1^st^ April 2020. Grocery stores were allowed to continue operations but with clear SOPs that restricted numbers that entered and left the site at a given time and the handling of trolleys. Food markets were allowed to continue operations. Factory operations and construction sites were restricted to only crucial employees and these were to camp around the factory area for the 14 days without being allowed to go home otherwise production and construction, respectively, were suspended for 14 days. Essential services like medical, agriculture and veterinary, telecommunication, door-to-door delivery, financial institutions, all media, private security companies, cleaning services, garbage collection, fire brigade, fuel stations, and water departments were allowed to continue operations. Cargo transport within Uganda and between Uganda and other countries by train, plane, lorries, pick-up trucks, “boda-bodas,” and bicycles was restricted to minimal numbers as follows: cargo—aircraft—only the crew; lorry—not more than 3 persons including the driver and his crew. Government employees were instructed to stay at home for the 14 days, except for the army, the police, health workers, and electricity, water, and telephone agencies. On the 31^st^ of March 2020, the president declared a nationwide curfew from 7:00 pm to 6:30 am prohibiting all movement except for cargo planes, lorries, pick-ups, and trains. “Boda-bodas” were instructed to stop operations at 2:00 pm. Saloons, lodges, and garages were shut for 14 days from the 1st of April 2020. Stay-at-home orders and all previous measures were sustained for another 21 days, starting 15^th^ April 2020 to 5^th^ May 2020.

A government response stringency index—a composite measure of the strictness of policy responses (such as mobility restrictions) over time, developed by the University of Oxford (Hale et al. 2021) was used as a measure for the strength of lockdown policies (including mobility restrictions) implemented by Uganda over time. The index on any given day is calculated as the average score of nine response (policy) indicators including school closures, workplace closures, restrictions on public gatherings, transport restrictions, stay-at-home requirements, and travel bans, each rescaled to a value from 0 to 100 (100 = strictest response). We obtained stringency index data for the period 15^th^ February 2020 to 10^th^ June 2021.

A phased opening of the country begun 4^th^ May 2020 with several restrictions relaxed and others lifted. In the first phase of opening, hardware shops, garages, warehouses, whole-sellers, metal and wood workshops, insurance providers, and legal services were allowed to re-open. Restaurants were allowed to resume operations but with only allowed take-away services. All the other measures announced earlier were stayed for another 14 days. The second phase of opening started on 26^th^ May 2022 where private cars were allowed to move but with only 3 persons and in only non-border districts. General merchandise shops excluding those in shopping malls, arcades, and food markets were re-opened but with strict social distancing. However, this relaxation of restrictions on private transport does not extend to the 40 border districts. Market vendors were relieved from camping at their stalls and were allowed to commute daily. Hotels and food restaurants were re-opened with social distancing and no air conditioning.

All other restrictions including closure of international borders and the airport, curfew from 7:00 pm to 6:30 pm, suspension of bars, night clubs, gyms, saunas, public swimming pools, and hair salons remained in place for another 21 days. On the 4^th^ of June 2020, public transport in non-border districts resumed but carrying half capacity. On 22^nd^ June 2020, capacity for private vehicles increased from 3 to 4 persons. “Boda-bodas” should continue with only the transportation of goods. Public and private transport was resumed in 33 of all border districts with no confirmed COVID-19 cases. On 20^th^ September 2020, the airport and land borders were opened for tourists, coming in and going out, provided they tested negative 72 h before arrival in Uganda and provided the tour operators ensured that the tourists did not mix with nationals. Returning Ugandans with negative PCR results were allowed to go home and followed by the country’s Ministry of Health. Restrictions on movements in border districts were lifted. Places of worship were opened but restricted to maxim 70 persons. Outdoor sports activities were re-opened with no spectators. However, curfew was sustained from 9:00 p.m to 6:00 a.m. and “boda-boda” movements were extended to end at 6:00 p.m. Casinos, other gambling centers, bars, cinemas, mobile markets, monthly cattle auction, and produce markets remained closed. Mass gatherings remained prohibited. On 15^th^ October 2020, schools were open to finalists, while on 1^st^ March 2021, schools were open to pre-finalists.

### Statistical analysis

All data processing and analysis was done using R version 4.1.1 (R Core Team 2021).

#### Effect of mobility restrictions on human movement and air quality

To determine the effect of mobility restrictions on human movement, we computed Pearson correlation coefficients between the government response stringency index and mobility in each of the six kinds of places tracked by Google and created scatter plots for the two cities (Kampala and Wakiso). Similarly, to determine the effect of mobility restrictions on air quality, we computed Pearson correlation coefficients between the government response stringency index and air quality and created scatter plots for the two cities (Kampala and Wakiso).

#### Association between air quality and human mobility

The association between air quality and human mobility was determined using Pearson correlation coefficients and visualized using scatter plots. We computed daily (24-h) averages for air pollutants (PM_2.5_ and PM_10_) for two cities (Kampala and Wakiso) that are well covered by air quality monitors and compared those with daily changes in mobility for Kampala and Wakiso. It is these districts that had complete mobility data relating to six types of places (grocery and pharmacy, parks, residential, retail and recreation, transit stations, and work places).

Multivariate analysis was performed using a linear model, adjusting for the government response stringency index. As a robustness check, Generalized Additive Models (GAMs) were fit to capture non-linearities in the data. For each district, we fit GAMs which were non-linear in mobility variables using the loess smoother and others with smoothing splines. We also fit models with both a linear and a non-linear government response stringency index. We compared the model fit for the linear models and the GAMs using the ANOVA test. GAMs were fit using the gam package in R Statistical Software (R Core Team 2021).

## Results and discussion

### Observed levels of air pollution in the two cities

**Fig. 1:**
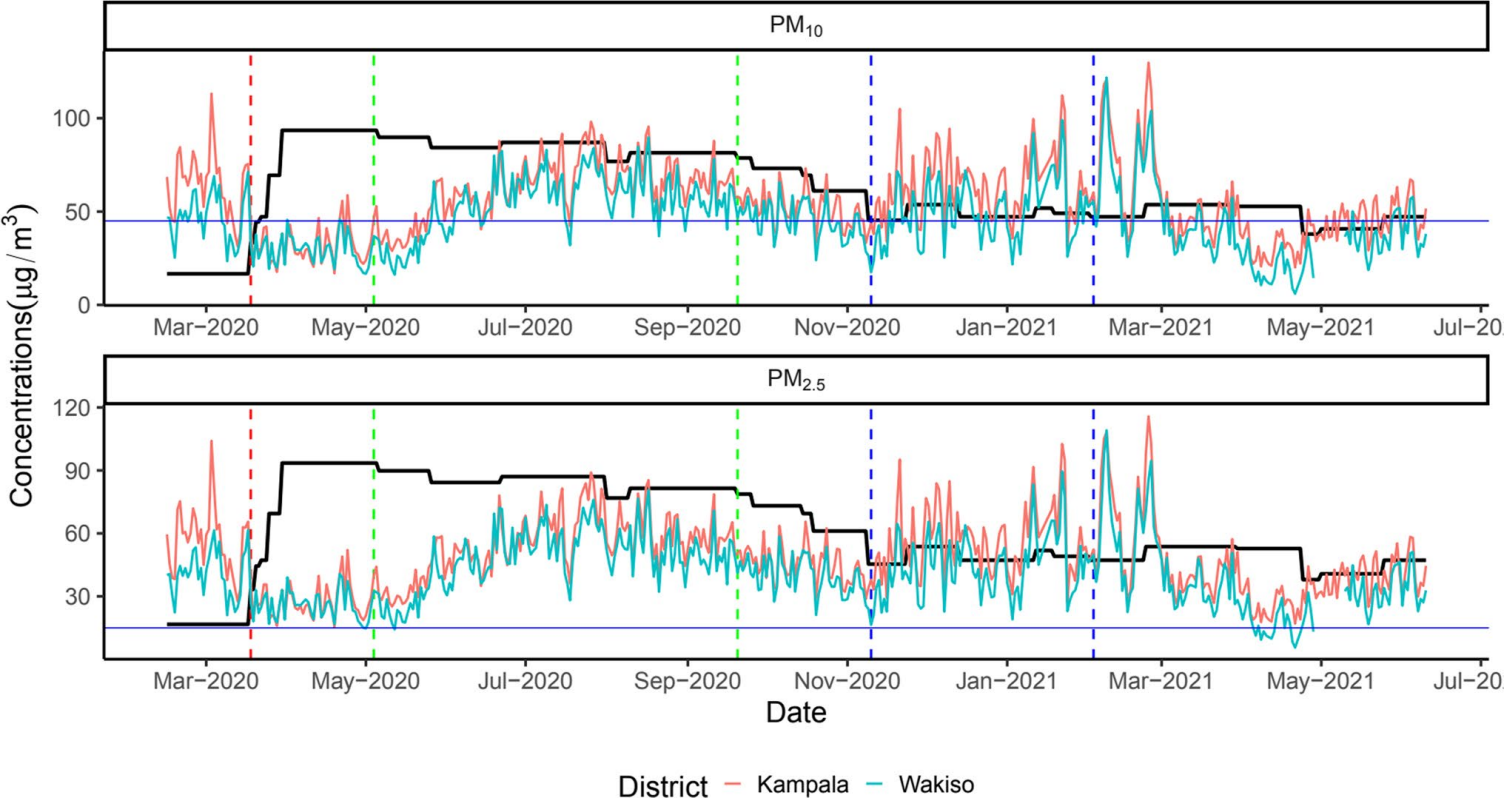
Overall ambient air quality temporal variation for Kampala and Wakiso districts

During the observation period (15^th^ February 2020 to 10^th^ June 2021), the 24-h average levels for fine particulate matter (PM_2.5_) in Kampala were consistently higher than the WHO recommended threshold (guideline limit) of 15 μg/m^3^ with a mean of 48.4 μg/m^3^ (range = 15.3–116.0), while the 24-h average PM_2.5_ levels for Wakiso fell below the WHO guideline limit in April 2021 with a mean of 40.5 μg/m^3^ (range = 5.6–109.0) (Fig. 1). On the other hand, the 24-average levels for coarse particulate matter (PM_10_) were lower than the WHO recommended threshold of 45 μg/m^3^ on some days in both cities with a mean of 55.5 μg/m^3^ (range = 16.9–130) for Kampala and 46.0 μg/m^3^ (range = 5.95–122.0) for Wakiso. The difference by city in mean levels of both PM_2.5_ and PM_10_ was not statistically significant ($p=0.2396$ for PM_2.5_ and $p=0.2396$ for PM_10_). The findings presented here confirm WHO reports that 91% of the world’s population live in places where air pollution levels exceed WHO guideline limits (WHO 2022). Although high levels of air pollution could indicate isolated air pollution events such as high pollution in city factories rather than depict mobility patterns at population level, here, we controlled for this by not including air quality data for sensors that showed irregular spikes at notable locations near factories in these locations.

The horizontal blue lines are the 24-h average levels recommended by WHO, i.e., 15 μg/m^3^ for PM_2.5_ and 45 μg/m^3^ for PM_10_. The red dashed vertical line indicates the date for start of restrictions in Uganda when mass gatherings were suspended, i.e., 18th March 2020. The first green dashed vertical line (from left to right) indicates the date (4th May 2020) for the start of the first phase of easing restrictions where whole sellers, metal and wood workshops, warehouses, insurance providers, and hardware shops were opened and restaurants were allowed to provide take-aways. The second green dashed line is the date (20th September 2020) when the country’s only International Airport and land borders were opened for tourists, restrictions on movements on border districts were lifted, and places of worship and sports activities were opened. The space between the dashed blue lines indicates the election period from 10th November 2020 when campaigns for presidential elections started to 3rd February 2021 when elections for subcounty/town/municipal division chairpersons and councilors were conducted. The black line indicates how government response stringency index varied over time during the observation period.

There was a very high positive correlation between PM_10_ and PM_2.5_ at all air quality observation sites (Supplementary Fig. 1), with all Pearson correlation coefficients greater than 0.99 and all $p$ values < 0.001. This is expected because PM_2.5_ is a subset of PM_10_. We therefore used PM_2.5_ in subsequent analyses determining the association between air quality and human mobility.

### How the lockdown affected mobility patterns and air quality

**Fig. 2:**
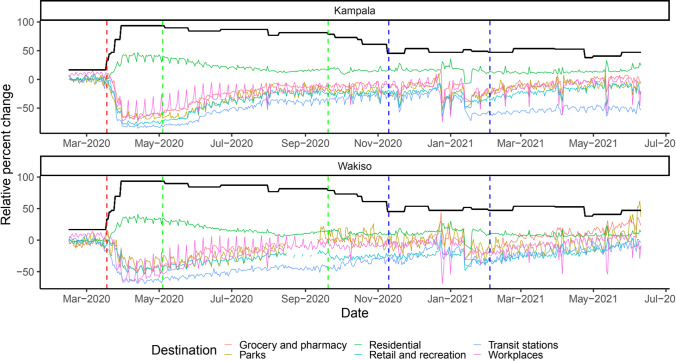
Change in mobility patterns in Kampala and Wakiso districts relative to the baseline

Institution of the first lockdown in the country on 18^th^ March 2020 led to an immediate increase in mobility in residential places and a decrease in mobility in non-residential places (grocery and pharmacy, parks, retail and recreation, transit stations, and work places) in both cities (Fig. 2). The increase in mobility in residential places due to institution of the lockdown is expected because lockdown restrictions included stay-at-home and work-from-home orders. The decrease in non-residential mobility demonstrates that lockdowns are an effective policy measure for decreasing human mobility so as to reduce the spread of infectious diseases like COVID-19. Without human movement, infectious particles would be less likely to be transferred from one person/location to another. Our findings are consistent with those of previous studies that found an increase in residential mobility and a decline in mobility in non-residential places during COVID-19 lockdown periods (Lawal and Nwegbu 2020; Sadowski et al. 2021; Saha et al. 2020; Saha et al. 2021). When a phased lifting of restrictions begun on 4^th^ May 2020, mobility in residential places reduced to values close to baseline values. The baseline is the median value, for the corresponding day of the week, during the five-week period 3^rd^ January 2020–6^th^ February 2020 (Google LLC 2021). On the other hand, mobility in non-residential places increased to close to baseline values.

The red dashed vertical line indicates the date (18th March 2020) for start of restrictions in Uganda when mass gatherings were suspended. The first green dashed vertical line (from left to right) indicates the date (4th May 2020) for the start of the first phase of easing restrictions where whole sellers, metal and wood workshops, warehouses, insurance providers, and hardware shops were opened and restaurants were allowed to provide take-aways. The second green dashed line is the date (20th September 2020) when the country’s only International Airport and land borders were opened for tourists, restrictions on movements on border districts were lifted, and places of worship and sports activities were opened. The space between the blue dashed lines indicates the election period from 10th November 2020 when campaigns for presidential elections started to 3rd February 2021 when elections for subcounty/town/municipal division chairpersons and councilors were conducted. The black line indicates how government response stringency index varied over time during the observation period.

Just like the case was with mobility, institution of a lockdown on 18^th^ March 2020 led to a reduction in air pollution levels in both cities with coarse particulate matter (PM_10_) values going below the recommended WHO threshold in March, April, and May 2020 (Fig. 1). The mean concentrations of PM_10_ reduced from 64.7 μg/m^3^ to 31.6 μg/m^3^ in Kampala and from 47.1 μg/m^3^ to 29.4 μg/m^3^ in Wakiso following the institution of a lockdown. Similarly, mean concentrations of PM_2.5_ reduced from 56.6 μg/m^3^ to 27.6 μg/m^3^ in Kampala and from 41.0 μg/m^3^ to 25.7 μg/m^3^ in Wakiso following the institution of a lockdown. These results are consistent with findings from other studies that showed an improvement in air quality following institution of lockdown measures in most cities around the world (Archer et al. 2020; Benchrif et al. 2021; Chauhan and Singh 2020, 19; Hernández-Paniagua et al. 2021; Sannigrahi et al. 2021; Yechezkel et al. 2021). However, just like the case was with mobility, these gains in air quality were short-lived and were promptly reversed when restrictions were lifted, i.e., following a phased start of lifting of restrictions on 4^th^ May 2020, air pollution levels increased to values above the recommended WHO thresholds until July 2020 onwards when air pollution started declining with PM_10_ values either reaching or falling below the recommended WHO threshold on some days. The mean concentrations of PM_10_ increased to 63.0 μg/m^3^ and 54.4 μg/m^3^ in Kampala and Wakiso, respectively, following the lifting of the lockdown. Similarly, mean concentrations of PM_2.5_ increased to 54.9 μg/m^3^ and 47.9 μg/m^3^ in Kampala and Wakiso, respectively, following the lifting of the lockdown.

**Fig. 3:**
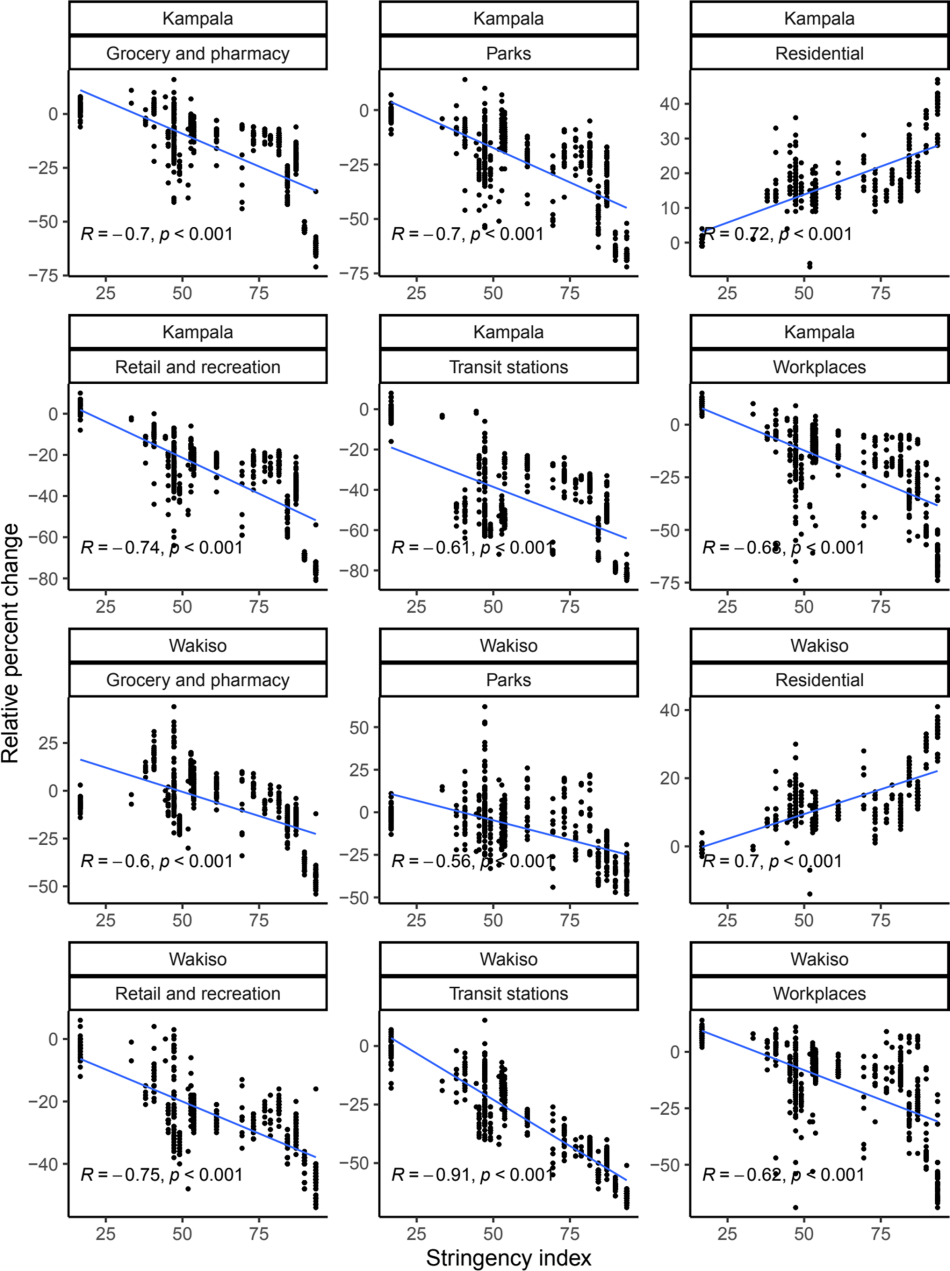
Correlation between changes in mobility (relative to the baseline) in Kampala and Wakiso districts and government response stringency index. The baseline is the median value, for the corresponding day of the week, during the five-week period 3rd January 2020–6th February 2020. Associated Pearson correlation coefficients ($R$) and a significance code for the $p$ value ($p$) are shown

For Kampala, the government response stringency index was negatively correlated with movement in grocery and pharmacy ($R = -0.70$, $p<0.001$), parks ($R = -0.70$, $p<0.001$), retail and recreation ($R = -0.74$, $p<0.001$), transit stations ($R = -0.61$, $p<0.001$), and work places ($R = -0.68$, $p<0.001$) and positively correlated with movement in residential places ($R = 0.72$, $p<0.001$) (Fig. 3). Similarly, for Wakiso, the government response stringency index was negatively associated with movement in grocery and pharmacy ($R = -0.6$, $p<0.001$), parks ($R = -0.56$, $p<0.001$), retail and recreation ($R = -0.75$, $p<0.001$), transit stations ($R = -0.91$, $p<0.001$), and work places ($R = -0.62$, $p<0.001$) and positively correlated with movement in residential places ($R = 0.70$, $p<0.001$) (Fig. 3).

**Fig. 4:**
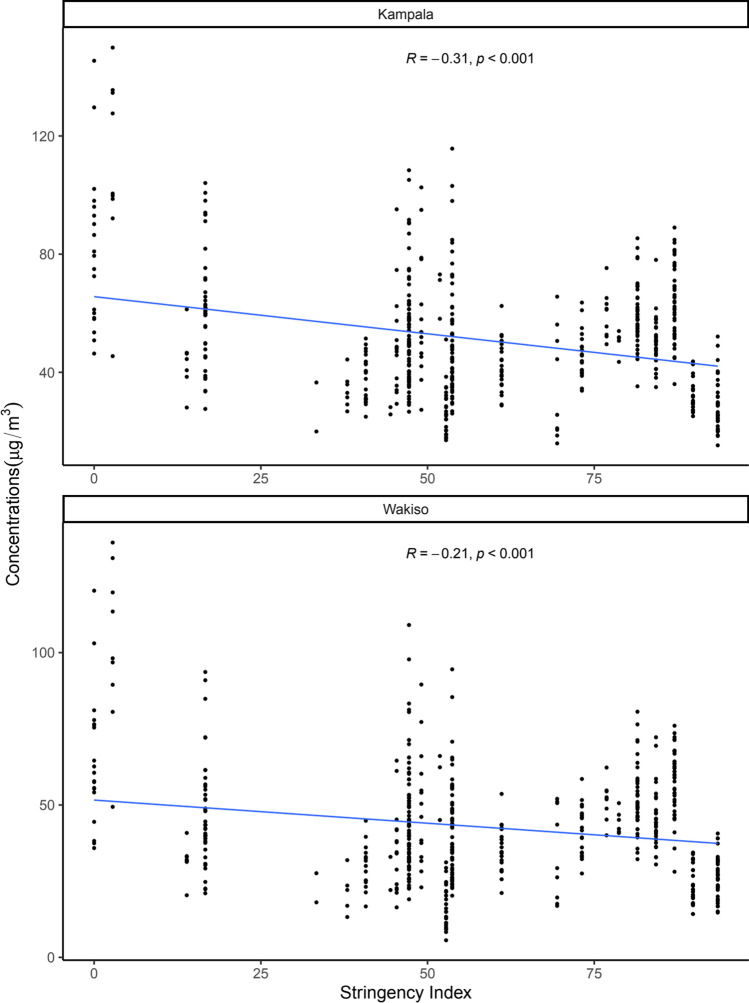
Correlation between fine particulate matter (PM_2.5_) in Kampala and Wakiso and government response stringency index. Associated Pearson correlation coefficients ($R$) and a significance code for the $p$ value ($p$) are shown

Similarly, air quality improved with stringency of restrictions in both Kampala and Wakiso districts, with $R = -0.31$ ($p<0.001$) and $R = -0.21$ ($p<0.001$), respectively (Fig. 4). Thus, as movement restrictions were tightened, people stayed more at home and movement in all other places reduced significantly leading to an improvement in air quality. This could be a result of reduced road vehicular traffic volume. The implication of this finding is that air quality closely mirrors movement data and thus may accurately depict movement patterns at population level.

### Association of air quality with human mobility

**Fig. 5:**
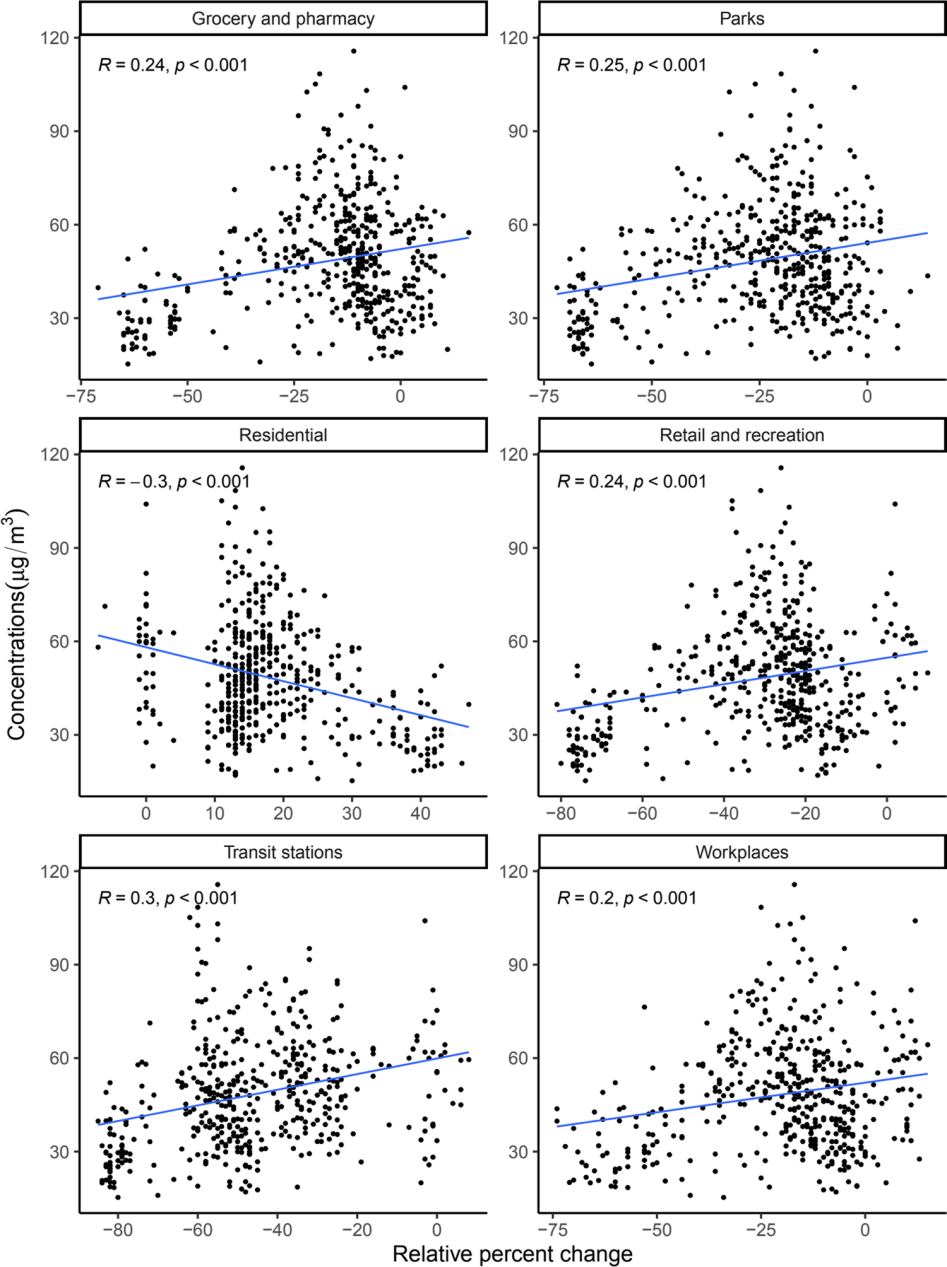
Correlation between changes in mobility (relative to the baseline) and fine particulate matter (PM_2.5_) in Kampala district. The baseline is the median value, for the corresponding day of the week, during the five-week period 3rd January 2020–6th February 2020. Scatter plots, associated Pearson correlation coefficients ($R$) and $p$ values ($p$) are shown

**Fig. 6:**
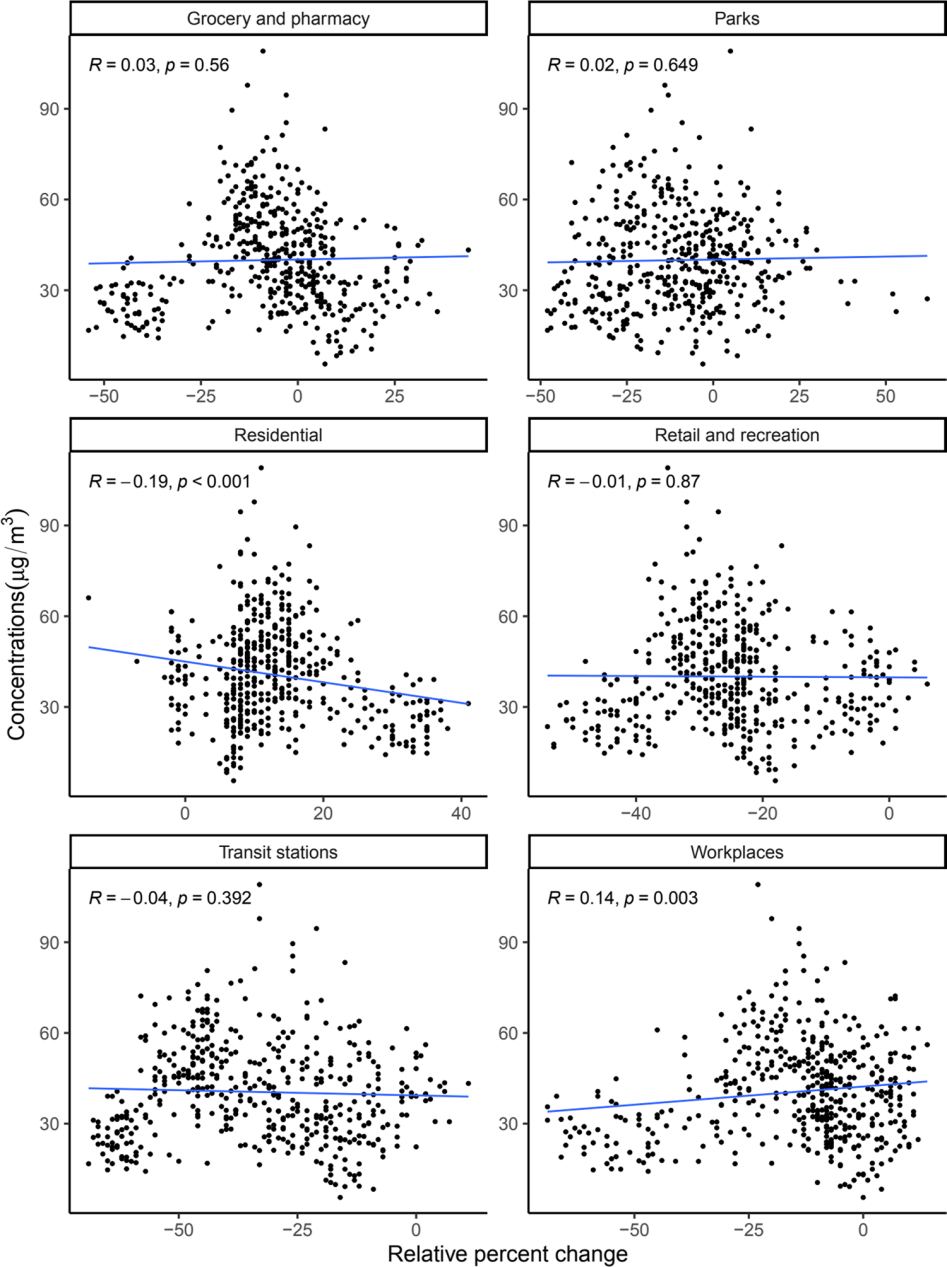
Correlation between changes in mobility (relative to the baseline) and fine particulate matter (PM_2.5_) in Wakiso district. The baseline is the median value, for the corresponding day of the week, during the five-week period 3rd January 2020–6th February 2020. Scatter plots, associated Pearson correlation coefficients ($R$) and $p$ values ($p$) are shown

In Kampala, air quality as measured by the amounts of atmospheric fine particulate matter (PM_2.5_) was positively associated with movement to groceries and pharmacies ($R = 0.24$, $p<0.001$), parks ($R = 0.25$, $p<0.001$), retail and recreation ($R = 0.24$, $p<0.001$), transit stations ($R = 0.3$, $p<0.001$), and work places ($R = 0.2$, $p<0.001$) and negatively correlated with movement within residential places ($R = -0.3$, $p<0.001$) (Fig. 5). These findings are consistent with those of a previous study in Barcelona that found a positive correlation between the mobility in places classified as retail and recreation with nitrogen dioxide and coarse particulate matter (Gorrochategui et al. 2021). Only associations between air quality and movement within workplaces and residential places were statistically significant in Wakiso, with $R = 0.14$ ($p<0.001$) and $R = -0.19$ ($p = 0.003$), respectively (Fig. 6).

In a multivariate analysis, air quality in Kampala was independently correlated with movement in retail and recreation (− 0.55; 95% CI =  − 1.009– − 0.099), parks (0.29; 95% CI = 0.033–0.543), transit stations (0.29; 95% CI = 0.156–0.424), workplaces (− 0.25; 95% CI =  − 0.43– − 0.079), and residential places in Kampala (− 1.02; 95% CI =  − 1.4– − 0.638) after controlling for the government response stringency index (Table 1). On the other hand, for Wakiso, only the correlation between air quality and movement in places of residence was statistically significant (− 0.99; 95% CI =  − 1.335– − 0.652) (Table 2). This could be because Wakiso is a more rural city compared to Kampala suggesting that air quality captures movement patterns more accurately in urban cities relative to less urban locations. This could also suggest higher compliance to movement restrictions in urban centers where enforcement is more concentrated in Uganda. This observation may also be attributed to increased domestic emissions due to people staying home more.

**Table 1:** Effect of changes in mobility on fine particulate matter (PM_2.5_) in Kampala district. Multivariate analysis using linear regression for the linear relationship between changes in mobility within the six kinds of places tracked by Google and air quality in Kampala district controlling for lockdown restrictions, i.e., the government response stringency index, with corresponding 95% confidence intervals (CIs) and significance codes for the $p$ values. Statistically significant $p$ values ($p<0.05$) are shown in bold font

Destination	Estimate	95% confidence interval	$p$ value
Retail and recreation	− 0.55	− 1.009– − 0.099	${\varvec{p}}\boldsymbol{ }<\boldsymbol{ }0.05$
Grocery and pharmacy	0.23	− 0.111–0.566	$p > 0.1$
Parks	0.29	0.033–0.543	${\varvec{p}}\boldsymbol{ }<\boldsymbol{ }0.05$
Transit stations	0.29	0.156–0.424	${\varvec{p}}\boldsymbol{ }=\boldsymbol{ }0$
Workplaces	− 0.25	− 0.43– − 0.079	${\varvec{p}}\boldsymbol{ }<\boldsymbol{ }0.01$
Residential	− 1.02	− 1.4– − 0.638	${\varvec{p}}\boldsymbol{ }=\boldsymbol{ }0$

**Table 2:** Effect of changes in mobility on fine particulate matter (PM_2.5_) in Wakiso district. Multivariate analysis using linear regression for the linear relationship between changes in mobility within the six kinds of places tracked by Google and air quality in Wakiso adjusting for lockdown restrictions, i.e., the government response stringency index, with corresponding 95% confidence intervals (CIs) and significance codes for the $p$ values. Statistically significant $p$ values ($p<0.05$) are shown in bold font

Destination	Estimate	95% confidence interval	$p$ value
Retail and recreation	− 0.12	− 0.356–0.113	$p > 0.1$
Grocery and pharmacy	− 0.15	− 0.333–0.025	$p < 0.1$
Parks	0.13	− 0.003–0.259	$p < 0.1$
Transit stations	− 0.04	− 0.302–0.213	$p > 0.1$
Workplaces	0	− 0.155–0.147	$p > 0.1$
Residential	− 0.99	− 1.335– − 0.652	${\varvec{p}}\boldsymbol{ }=\boldsymbol{ }0$

The results of ANOVA tests comparing linear models with GAMs (Supplementary Figs. 2–5) provided statistical evidence to suggest that incorporating nonlinear relationships of the mobility variables did not improve the model, i.e., the linear model provided a better fit compared with the GAMs with $p < 0.001$ for both Kampala and Wakiso.

## Conclusions

Beyond the direct counting of moving persons, several analogs could be used to represent quantification of human mobility, including road vehicular traffic volume, mobile phone tracking, and fuel consumption. However, collecting such data on a city or district wide scale would not only be prohibitively expensive in money and time, it would also offer low resolution and sensitivity. Mobile phone tracking comes closest but is also limited by the relative paucity of mobile phones with enabled GPS location devices in developing countries, the ability of users to turn off GPS location, and the rather arduous and lengthy process of obtaining such data from mobile phone companies.

We observed pollution levels for fine particulate matter (PM_2.5_) that were above WHO guideline limits in both cities in Uganda even during COVID-19 lockdowns despite some improvement in air quality during these periods. Thus, more work needs to be done to address the problem of air pollution in this setting as well as other cities around the world. The institution of restrictions also led to an increase in residential mobility and a decrease in non-residential mobility. Thus, lockdowns are an effective way of reducing human mobility to interrupt infectious disease transmission as well as improve air quality.

Institution of a lockdown in the country on 18^th^ March 2020 led to an increase in mobility in residential places and a decrease in mobility in non-residential places in both cities. Similarly, institution of a lockdown led to a reduction in air pollution levels in both cities. When a phased lifting of restrictions begun on 4^th^ May 2020, mobility in residential places reduced to values close to baseline values and air pollution levels increased. Air quality in both cities improved with stringency of movement restrictions which was positively correlated with movement in residential places and negatively correlated with movement in non-residential places.

Furthermore, in a multivariable analysis, air quality was independently positively correlated with movement in non-residential places and negatively correlated with residential mobility in Kampala and Wakiso. Taken together with growing evidence from a literature review on the topic, these findings suggest that air quality data closely mirrors human mobility data and could thus be used as a proxy to human movement patterns in these places. Infectious disease control programs could thus leverage air quality data to study transmission patterns for infectious diseases and inform control measures without compromising on the privacy of individuals and other limitations that are associated with the use of location information from mobile phones.

## Supplementary information

Below is the link to the electronic supplementary material.

Supplementary file1 (DOCX 204 KB)

## Data Availability

Human mobility data is from Google Community Mobility Reports and is available from Google as a free download (https://www.google.com/covid19/mobility/). COVID-19 case data is available from the World Health Organization (https://covid19.who.int/table). Air Quality data is available upon submitting a request at the AirQo project website (https://www.airqo.net/). The datasets generated during and/or analyzed during the current study are available from the corresponding author on reasonable request.
