# Supplementary material for: Air pollution and mobility patterns in two Ugandan cities during COVID-19 mobility restrictions suggest the validity of air quality data as a measure for human mobility
Source: Environ Sci Pollut Res Int. 2022 Dec 15;30(12):34856–71. doi: 10.1007/s11356-022-24605-1 (PMC9751517; doi:10.1007/s11356-022-24605-1)
Supplement: Supplementary file 1 — Supplementary file1 (DOCX 204 KB) [file 11356_2022_24605_MOESM1_ESM.docx]

**
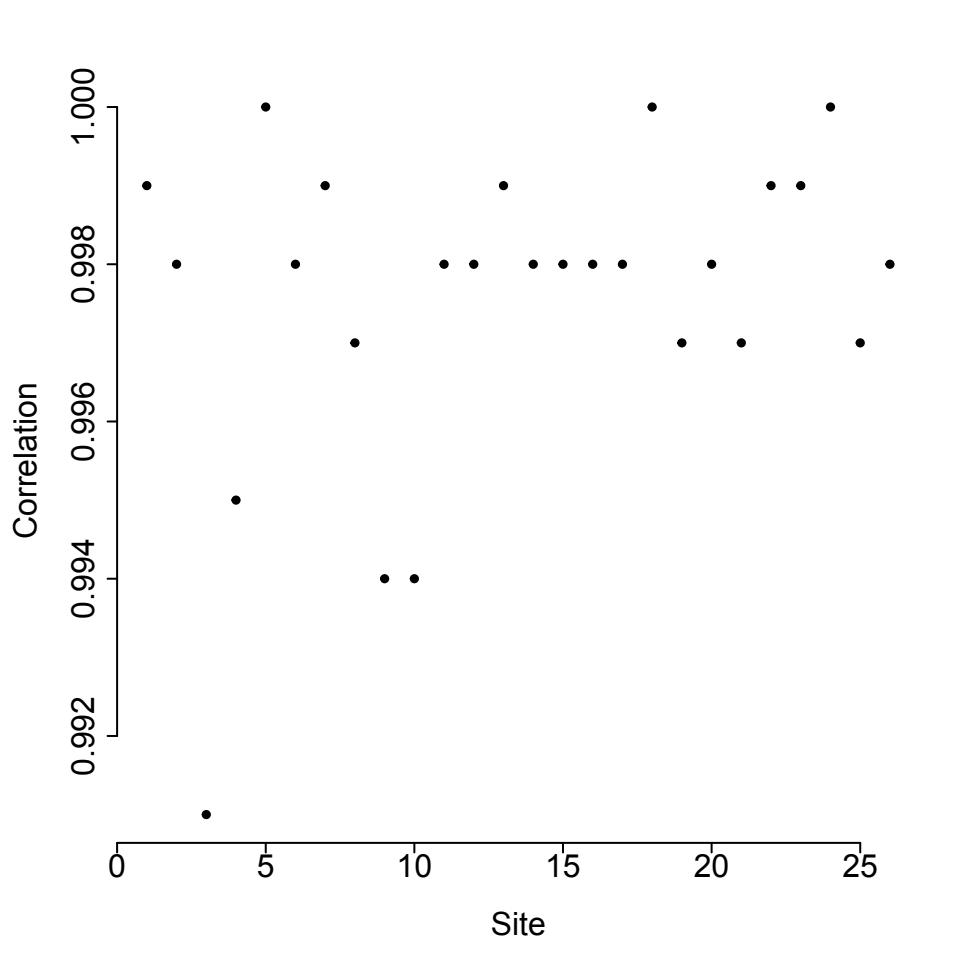
Supplementary Fig. 1** Correlation (Pearson correlation) between PM_2.5_ and PM_10_ at sites

**
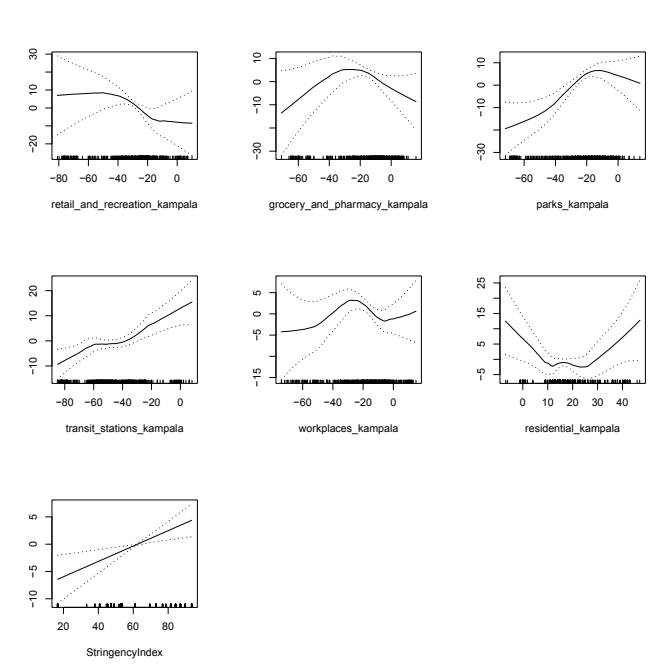
Supplementary Fig.** 2 Generalized Additive Model (GAM) with Loess Smoother for non-linearity of mobility variables (Kampala)

**
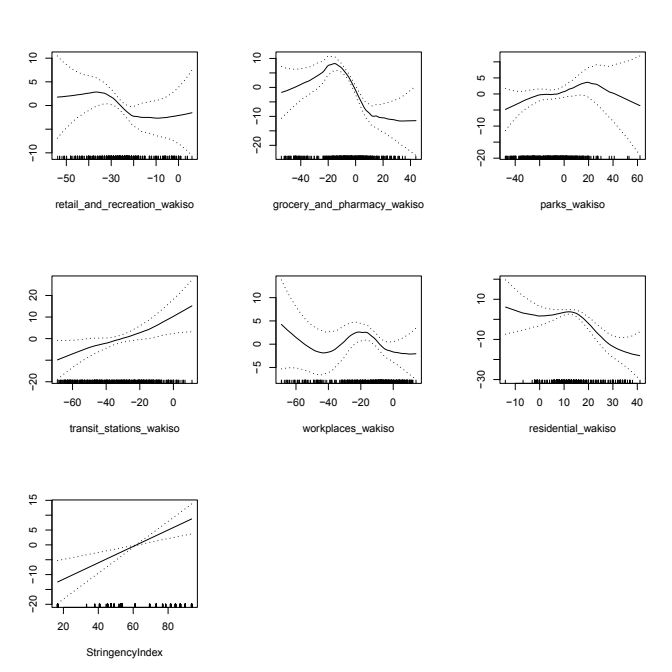
Supplementary Fig. 3** Generalized Additive Model (GAM) with Loess Smoother for non-linearity of mobility variables (Wakiso)

**
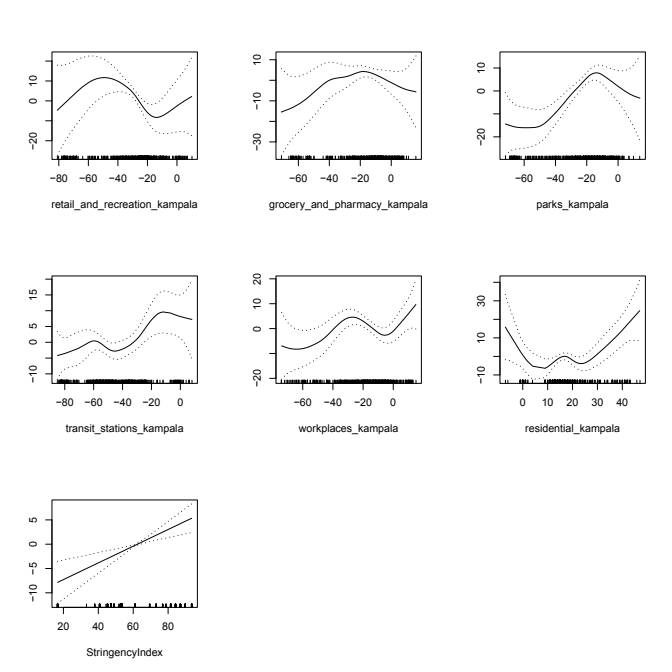
Supplementary Fig. 4** Generalized Additive Model (GAM) with Smoothing Splines for non-linearity of mobility variables (Kampala)

**
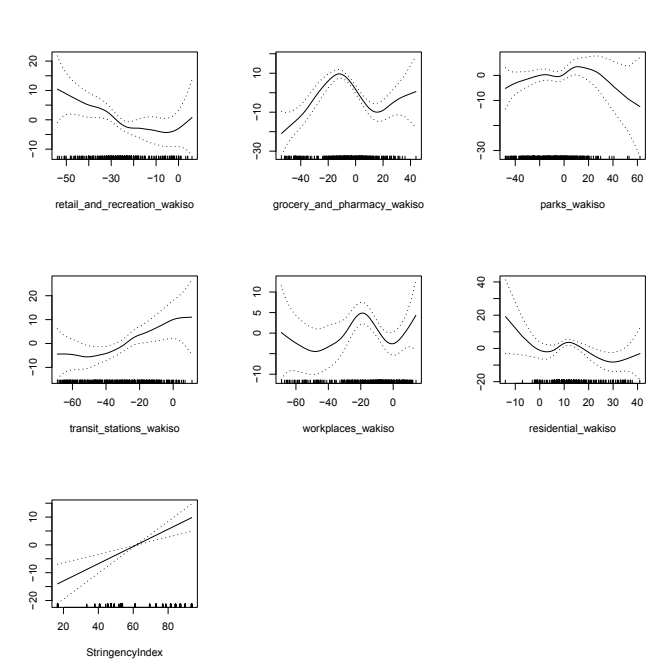
Supplementary Fig. 5** Generalized Additive Model (GAM) with Smoothing Splines for non-linearity of mobility variables (Wakiso)
